# Carbapenem-Resistant *Pseudomonas aeruginosa* Strains-Distribution of the Essential Enzymatic Virulence Factors Genes

**DOI:** 10.3390/antibiotics10010008

**Published:** 2020-12-24

**Authors:** Tomasz Bogiel, Małgorzata Prażyńska, Joanna Kwiecińska-Piróg, Agnieszka Mikucka, Eugenia Gospodarek-Komkowska

**Affiliations:** Microbiology Department, Ludwik Rydygier Collegium Medicum in Bydgoszcz, Nicolaus Copernicus University in Toruń, 9 Maria Skłodowska-Curie Street, 85-094 Bydgoszcz, Poland; malgorzata_szabelska@wp.pl (M.P.); j.kwiecinska@cm.umk.pl (J.K.-P.); a.mikucka@cm.umk.pl (A.M.); gospodareke@cm.umk.pl (E.G.-K.)

**Keywords:** carbapenems, carbapenem-resistant *Pseudomonas aeruginosa*, *Pseudomonas aeruginosa*, *Pseudomonas aeruginosa* genotypes, *Pseudomonas aeruginosa* virulence, resistance to carbapenems, virulence, virulence factor genes

## Abstract

*Pseudomonas aeruginosa* is one of the most commonly isolated bacteria from clinical specimens, with increasing isolation frequency in nosocomial infections. Herein, we investigated whether antimicrobial-resistant *P. aeruginosa* strains, e.g., metallo-beta-lactamase (MBL)-producing isolates, may possess a reduced number of virulence genes, resulting from appropriate genome management to adapt to a changing hospital environment. Hospital conditions, such as selective pressure, may lead to the replacement of virulence genes by antimicrobial resistance genes that are crucial to survive under current conditions. The study aimed to compare, using PCR, the frequency of the chosen enzymatic virulence factor genes (alkaline protease-*aprA*, elastase B-*lasB*, neuraminidases-*nan1* and *nan2*, and both variants of phospholipase C-*plcH* and *plcN*) to MBL distribution among 107 non-duplicated carbapenem-resistant *P. aeruginosa* isolates. The gene encoding alkaline protease was noted with the highest frequency (100%), while the neuraminidase-1 gene was observed in 37.4% of the examined strains. The difference in *lasB* and *nan1* prevalence amongst the MBL-positive and MBL-negative strains, was statistically significant. Although *P. aeruginosa* virulence is generally more likely determined by the complex regulation of the virulence gene expression, herein, we found differences in the prevalence of various virulence genes in MBL-producers.

## 1. Introduction

*Pseudomonas* spp. are typically opportunistic pathogens implicated in a wide range of plant, animal, and human infections. The most important and the most often isolated representative is *Pseudomonas aeruginosa*, with its increasing role in the nosocomial infections (e.g., respiratory and urinary tract, skin and soft tissues, ear, eye, and bacteremia) [1]. *P. aeruginosa* has intrinsic resistance to numerous antimicrobial agents and also easily acquires resistance to many antibiotics, including carbapenems [2]. These beta-lactams (e.g., imipenem and meropenem) are often the last resort antibiotics. *P. aeruginosa*’s resistance to carbapenems usually results from the acquisition of genes coding for carbapenemases (e.g., metallo-beta-lactamase (MBL)—class B carbapenemases). It has been shown previously that the most common *P. aeruginosa* carbapenemases in Poland are VIM- and IMP-like enzymes and that the incidence of carbapenem-resistant *P. aeruginosa* (CRPA) strains and their contribution to infections are increasing [1,3,4,5]. It is worth mentioning that is has been confirmed that CRPA strains with some dominant sequence types may efficiently spread clonally [6]. Moreover, new types of carbapenem resistance (e.g., IMP, NDM, DIM and PME β-lactamases) amongst *P. aeruginosa* strains are detected in Europe [5], which is proof of the ongoing evolution of these bacteria. It is noteworthy that some interesting recent findings on complex CRPA strains resistance, also in Poland, have been made [5].

The pathogenicity of *P. aeruginosa* is determined by the synthesis of several cell-associated compounds (e.g., flagella, pili, alginate capsule, and lipopolysaccharide). However, *P. aeruginosa* is also capable of secreting, on a large scale, proteins with potential roles in pathogenicity—usually extracellular factors (mostly toxins, e.g., exotoxin A and exoenzymes) and enzymes (e.g., proteases, elastases, phospholipase C, and neuraminidases) [7]. They usually contribute to the *P. aeruginosa* colonization of the host cells. The phospholipase C gene (*plcH*) encodes a hemolytic toxin that destroys cell membranes and hydrolyzes phospholipids, especially phosphatidylcholine, thus hemolyzing erythrocytes and destroying surfactant lipids [7,8]. *P. aeruginosa* may also possess the *plcN* gene that encodes a non-hemolytic counterpart of phospholipase C, which is 40% identical in protein structure and hydrolyzes phosphatidylserine and phosphatidylcholine [8]. Elastase B (LasB), encoded by the *lasB* gene, is a zinc metalloprotease that destroys the structural proteins of the cells (e.g., collagen, non-collagen proteins, and elastin) [7,9]. Neuraminidases (−1 and −2), encoded by the *nan1* and *nan2* genes, respectively, are the extracellular protein enzymes believed to play an essential role in bacterial implantation, adhesion to host cells, and infection process, usually in the respiratory tract [10]. Alkaline protease (AprA—encoded by the *aprA* gene) is a zinc metalloprotease that degrades many crucial proteins, like gelatin, casein, and complement factors produced by the human cells. It also interferes with the immune system by inhibiting complement activation, neutrophil phagocytosis, and fibronectin, thus promoting survival in the host [7]. Moreover, AprA, contributes to the increased virulence of the strains, e.g., pyocyanin overproduction in the presence of particular proteins or peptides [11]. All protein virulence factors are chromosomally-encoded, and their gene sequences and positions are well known from the *P. aeruginosa* PAO1 strain (earlier called “*Pseudomonas aeruginosa* strain 1”) genome sequence [12].

The co-existence of antimicrobials resistance determinants and virulence factors in *P. aeruginosa* isolates is an alarming threat. It underlines the importance of monitoring multidrug-resistant pathogen in order to determine optimal therapeutic options against such infections [13].

The aim of this study was to determine the genetic features of 107 non-repeatable CRPA strains by assessing the distribution of six genes encoding enzymatic virulence factors. Herein, we tested the hypothesis that strains of CRPA will vary in the carriage of certain virulence genes based on their carbapenem resistance (expression of MBLs) type.

## 2. Results

MBLs, found with the highest frequency in *P. aeruginosa* isolates, are Verona Integron-encoded MBL (VIM) and IMiPenemase (IMP) [1,2]. The genes coding for MBLs were detected in 32 (29.9%) of the tested strains. *bla*_VIM_ genes were exclusively detected in the strains, but none of the examined strains were positive for the presence of the *bla*_IMP_ gene, suggesting other resistance mechanism than one of the mentioned MBLs. Minimal Inhibitory Concentration (MIC) values for each of the examined strain were >4 and/or >8 mgl/L for imipenem and meropenem, respectively.

The prevalence of selected virulence genes varied broadly between CRPA strains: the *aprA* gene was noted in all of the tested strains, whereas *nan1* was observed with the lowest frequency in 37.4% of the examined isolates. The distribution of the six virulence genes grouped each strain into one of the nine observed genotypes (A–I), with a different virulence genes set. Their prevalence and the examined genes distribution amongst the examined strains are shown in Table 1. The most common (47.7%) was genotype A, including *aprA*, *plcH*, *plcN*, *las B* and *nan2* genes.

The differences in the virulence gene frequency between MBL-positive and MBL-negative CRPA strains are presented in Table 2.

The distribution of the examined virulence factor genes with respect to specimen type that the strains were isolated from in a number higher than 5 is presented in Table 3.

The strains isolated from respiratory tract secretion and blood-derived samples, on the genetic level, presented the highest virulence potential—the highest percentages of different virulence gene were observed among them. However, the differences in gene distribution in terms of the type of clinical specimen that the carbapenem-resistant strains were isolated from, observed in the present study, were not statistically significant.

## 3. Discussion

*P. aeruginosa*’s genome plasticity was recently confirmed by Ramsay et al. [14], showing that the environmental isolates are mostly susceptible to antimicrobials while their nosocomial counterparts rather resistant to antibiotics, possibly due to an adaptation to a hospital environment. The environmental antimicrobial susceptible *P. aeruginosa* strains are also infectious for human but it is likely that they become resistant during the infection, possibly with the influence of antimicrobial therapy pressure [14]. A growing number of reports on the increased virulence of multi-drug resistant *P. aeruginosa* isolates, when compared to more susceptible strains, can be found in the available literature [15,16,17,18,19].

It is known that the expression of *P. aeruginosa* virulence factors is a very complex process. It depends not only on the manner of the synthesized virulence factor but also on the antimicrobial resistance mechanism type, as was recently found [20]. It has also been recently shown that particular resistance mechanisms can easily and favorably co-exist with virulence genes in particular sets of strains [13,21,22]. Other authors, on the contrary, have suggested that the virulence of antibiotic-resistant *P. aeruginosa* strains (including carbapenem-resistant isolates) has somehow been reduced [16,17,18,19]. One of the explanations of this phenomenon is that bacterial cells somehow selectively activate genes, when necessary from the survival point of view, while silencing other ones. This phenomenon has been previously observed, e.g., during bloodstream infection [23,24]. In this scenario, the successful treatment of the infections caused by them would depend not only on the appropriate antimicrobials usage but also on the downregulation of virulence gene expression.

The second explanation for the reduced virulence of the strains is due to bacterial genome “management” that would allow them to survive in unfavorable conditions, e.g., antibiotic pressure in a hospital environment. Virulence genes would be then “replaced” by antimicrobial resistance genes that are crucial. It would be fully understood and justified but has not yet been confirmed. The hypotheses of *P. aeruginosa* genome evolution was discussed previously [25]. However, without more detailed study, the mechanism of virulence gene loss (e.g., rearrangement or common genes deletions) cannot be elucidated.

In the available literature, numerous researchers have characterized genetic features of *P. aeruginosa* with respect to different variables (e.g., strain origin, clinical specimen, and patients’ hospitalization length). However, little information on virulence gene frequency in CRPA strains can be found in the relevant literature [26].

It is noteworthy that a high level of virulence gene carriage was observed in our study—only 3% of the examined strains possessed less than four of the selected virulence genes, while the corresponding values for five and six genes were 50.5% and 33.6%.

All of the examined CRPA strains possessed *aprA* genes. This result confirmed previous findings made by Rojo-Bezares et al. [27], as well as the results obtained by Tingpej et al. [28] regarding AprA’s role as one of the fundamental *P. aeruginosa* virulence factors, the presence of which additionally results in the expression of other virulence factors [11]. In the results of the present study, all the examined strains, including urine-derived isolates, possessed the *aprA* gene. A relatively lower percentage of *aprA*-positive strains (82.5%) was noted by Liew et al. [18], while the lowest level (16.6%) was observed by Sabharwal et al. [29]. It is noteworthy that the results of the latest were based on a small number of 12 strains only.

In the available literature, little is known on *nan1* and *nan2* genes distribution amongst *P. aeruginosa* strains, with no information on the prevalence of these genes amongst CRPA. In the current study, neuraminidase-1 and -2 encoding genes were observed in 37.4% and 87.9% of the strains, respectively. Lanotte and co-authors [30] noted the corresponding values in 53.1% and 100% of strains, but not for CRPA isolates. The presence of the neuraminidase-1 gene was observed by the mentioned authors with the lowest frequency in urine-derived strains, similarly to our own results. According to Strateva et al. [31], the neuraminidase-1 gene was found amongst 38.2% of the examined *P. aeruginosa* strains (not CRPA exclusively). This value seems to be the closest to the results obtained in our study.

In the current study, the *nan1* gene was disseminated with the highest frequency among the strains cultured from respiratory tract secretion and blood samples, while the *nan2* gene was the most frequently observed amongst the strains derived from wound swabs and blood-derived isolates. Statistically significant differences were observed for *nan1* gene frequency, when compared to the rest of the detected genes (Table 1). Though the *nan1* gene was observed with the lowest frequency in the present study (amongst 37.4% of the examined strains only), its presence was observed at an almost three-fold higher level in MBL-negative strains when compared to the MBL-positive counterparts, 46.7% vs. 15.6%, respectively. This difference in the distribution of the *nan1* gene between MBL-negative and MBL-positive strains was statistically significant (*p* = 0.048) (Table 1). It is noteworthy that it is believed to be the evidence of the reduced virulence factor gene carriage in carbapenem-resistant, MBL-positive *P. aeruginosa* strains.

According to Lanotte et al. [30], both phospholipase-coding genes were found in all of the strains included in their studies. The results of the presented study confirmed their results, indicating presence of the mentioned genes amongst a high percentage (99.1%) of the examined strains. These findings were also close to the recent results of the study conducted by Pournajaf et al. [32] and Ellappan et al. [13], showing the *plcH* gene in 96.5% and 92% of the tested strains, respectively. In turn, they were in contrast to the results of the study conducted by the first mentioned author [32], where the *plcN* gene was found only in 32.8% of the stains included in their research. To our knowledge, this is the lowest percentage of phospholipase gene presence that was noted at a similar level (45%) only by Fazeli and Momtaz [33]. Wolska and Szweda [34], depending on the strain origin subgroups, reported 76.9–100% and 76.9–91.8% of *plcH* and *plcN* gene carriage amongst *P. aeruginosa* strains, respectively. Similar results were obtained by Faraji et al. [35], reaching 87.7% and 60% of *plcH*/*plcN* genes in a group of strains derived from the specimens collected from cystic fibrosis patients and the corresponding values of 79% and 63.1% amongst the strains cultured from the specimens derived from burn patients.

*lasB* genes, according to Lanotte et al. [30], Rojo-Bezares et al. [27], Tingpej et al. [28], and Pournajaf et al. [32], were found in all of the strains included in their studies. Similar results were obtained by Mitov et al. [36] regardless of the patients’ conditions—with or without cystic fibrosis. The latter case referred to the examined *P. aeruginosa* strains derived from specimens of cystic fibrosis patients, and LasB significance in chronic infections caused by *P. aeruginosa* was confirmed. The existence of this virulence gene was noted by Ellappan et al. [13], Liew et al. [18], and Sabharwal et al. [29] at the levels of 94%, 83.3%, and 75%, respectively. Wolska and Szweda [34] reported 84.6–100% of the *P. aeruginosa* strains carried the *lasB* gene with respect to isolates subtypes. The results of our study confirmed their findings, indicating the presence of the mentioned gene amongst 91.6% of the tested isolates. Surprisingly, to our knowledge, the lowest level of elastase B gene presence (18%) among the tested *P. aeruginosa* strains was noted by Dehbashi et al. [21] in a study performed for the strains derived from clinical specimens other than those of respiratory tract origin.

Genes for *lasB* were detected in all the examined *P. aeruginosa* strains isolated from urine and respiratory tract secretions, which might confirm the necessity of this virulence gene’s presence in the pathogenesis of urinary and respiratory tract infections. In our study, *lasB* gene frequency amongst MBL-positive strains was reduced by 19.2% when compared to their MBL-negative counterparts. This difference in *lasB* gene frequency was statistically significant.

In the present work, apart from *aprA*, *plcH* and *plcN*, *lasB* was noted with the highest frequency among the examined strains, regardless of the specimen that the isolates were derived from. This was a very important finding because it might have some potential in terms of the application of the mentioned genes as universal *P. aeruginosa* presence markers, e.g., to establish DNA amplification-based diagnostic tests.

It is noteworthy that the statistical analysis of the results revealed some undoubted regularities observed in the present study. The most important observation and statistically significant difference was found between the dissemination of the *lasB* (*p* = 0.0038) and *nan1* genes (*p* = 0.048). Amongst the separate two subgroups of the strains, MBL-positive and MBL-negative, *lasB* was found for 78.1% vs. 97.3%, respectively, while the corresponding values for *nan1* gene were 15.6% vs. 46.7%, respectively. This is believed to be the first evidence of the reduced virulence factor gene carriage in carbapenem-resistant MBL-positive *P. aeruginosa* strains with respect to MBL production ability. The lowest incidence of the *nan1* gene (37.4%), when compared to the rest of the virulence genes detected in the present research, also revealed statistical importance (*p* = 0.0134). There was also a statistically significant correlation in the co-existence of *lasB* and both phospholipases genes, which were altogether found in almost all of the examined strains.

Taken together, a great diversity could unquestionably be observed amongst CRPA strains. This may have resulted from their phenotypic properties, like different antimicrobial susceptibility patterns, including to carbapenem and other antibiotics or chemotherapeutics. This variety was also based on the existence of particular set of plasmid- or chromosomally-encoded genes within particular strains groups. The diversity could also appear as different virulence gene prevalence amongst strains derived from different clinical specimens. The cause of this diversity might be genetic material reorganization, such as obtaining and maintaining of the genes crucial for survival in a hospital environment (e.g., carbapenem-resistance genes) and a loss of the currently unnecessary virulence factor-coding genes, e.g., as observed for *nan1* and *lasB* genes. In summary, this study reported what is believed to be the first evaluation of reduced virulence factor gene carriage in CRPA strains, with respect to MBL gene presence. Further investigations are required to understand the mechanism of *P. aeruginosa* virulence and the involvement of other contributing factors. A standard procedure of the virulence genes detection in microbiological laboratory would provide tools for the rapid diagnostic monitoring of virulent strain presence and their spread to help in establishing of better novel (molecular) strategies for the treatment of the infections caused by *P. aeruginosa* [37] or, at least, their control.

## 4. Materials and Methods

### 4.1. Bacterial Strains and Their Selection Criteria

Initially, the study involved 119 clinical isolates of imipenem- and meropenem-resistant *P. aeruginosa*. All of them were derived from the collection the Microbiology Department Ludwik Rydygier Collegium Medicum in Bydgoszcz Nicolaus Copernicus University in Toruń. The isolates were mostly derived from bronchoalveolar lavage (22.7%), urine (21.9%) and wound swab samples (16.8%). Every strain was isolated from a different patient. The majority of them were hospitalized in an anesthesiology and intensive care clinic (34.5%) and different surgery clinics (25.2%). Though every strain was isolated from a different patient, after the genotyping screening of the DNA similarity of the isolates, performed using the Polymerase Chain Reaction-Random Amplified Polymorphic DNA (PCR-RAPD) technique [38], 12 strains were considered repeatable and excluded from further studies (data not shown). Thus, the prevalence of virulence and carbapenemase genes was evaluated for the remaining number of 107 non-repeatable *P. aeruginosa* isolates.

### 4.2. Strain Identification

*P. aeruginosa* identification was done based on the growth on simple media in aerobic conditions, selective growth on a cetrimide-supplemented medium (*bio*Mérieux, Craponne, France), and the ability to produce dyes. The final identification was confirmed in Vitek2 Compact system using GN ID cards (*bio*Mérieux, Craponne, France). *P. aeruginosa* strain derived from American Type Culture Collection (ATCC 27853) was used for identification test quality control.

### 4.3. Bacterial Genomic DNA Isolation

The Genomic Mini Kit (A&A Biotechnology, Gdynia, Poland) was applied for DNA isolation, according to the manufacturer’s protocol. In order to confirm the DNA isolation accuracy and to avoid false-negative results, the concentrations of all DNA samples were initially checked spectrophotometrically (Photometer, Eppendorf, Germany). DNA samples were then unified in terms of their concentration for PCR-RAPD application and stored afterwards at 4 °C before further use in PCR for MBL gene detection and the presence of the virulence genes.

### 4.4. Detection of MBLs and MBL Genes

All of the examined strains were initially checked for the presence of the MBL-type carbapenemases phenotypically, using the method called the double disc synergy test with the imipenem-EDTA and the ceftazidime-MPA discs, described by Lee et al. [39]. All of the strains were also checked for the MBLs presence using the method described by Yong et al. [40]. Among the tested strains, only these with *bla*_VIM_ gene were all positive for MBLs detection, regardless of the methodology applied (data not show). They are called MBL-positive, while the rest - MBL-negative.

A PCR assay was performed to detect the genes for the most common *P. aeruginosa* carbapenemases in Poland—the VIM- and IMP-like enzymes. It was performed in a PCR duplex version using primers 5′-GTT TGG TCG CAT ATC GCA AC-3′ and 5′-AAT GCG CAG CAC CAG GAT AG-3′ for *bla*_VIM_ and 5′-GAA GGY GTT TAT GTT CAT AC-3′ and 5′-GTA MGT TTC AAG AGT GAT GC-3′ for *bla_I_*_MP_, as previously described [41] for all of the examined isolates. PCR reaction products were separated by electrophoresis on 1% agarose gel in a 1 × concentrated Tris/Borate/EDTA running buffer (TBE) at 9 V/cm for one hour in a MINI SUB^TM^ DNA CELL (Bio-Rad, Feldkirchen, Germany) apparatus. Their visualizations were recorded and documented in Gel Doc 2000 system using Quantity One (Bio-Rad, Feldkirchen, Germany) program. *P. aeruginosa* strains carrying the *bla*_IMP_ or *bla*_VIM_ genes and the ATCC 27853 strain served as the positive and the negative controls of PCR, respectively.

### 4.5. Virulence Factor Genes Detection

The presence of 6 virulence factor genes was determined by PCR in a separate reaction for each gene. The genes were amplified with primers selected on the basis of the published PAO1 strain genome sequence [12] (Table 4). The amplification procedure was carried out as previously described by Lanotte et al. [30] and Finnan et al. [42] in 0.2 mL test tubes (Eppendorf) with a final volume of 20 μL. Briefly, *Taq* polymerase was used with total activity of 1 U per reaction in a 1 × concentrated polymerase buffer with MgCl_2_ at a final concentration of 1.5 mM (Go *Taq* G2 Polymerase, Promega, Walldorf, Germany, FirePol DNA Polymerase, Solis BioDyne, Tartu, Estonia) and deoxynucleotide triphosphates (dNTPs) set at a final concentration of 200 μM (Promega, Walldorf, Germany, Solis BioDyne, Tartu, Estonia). Primers were used at the final amount of 12.5 pmol per reaction (Integrated DNA Technologies, Coralville, IA, USA, Genomed, Warszawa, Poland). Their sequences for different genes are shown in Table 4. The isolated DNA samples were added subsequently. DNA isolated from the *P. aeruginosa* PAO1 strain, kindly provided by National Medicines Institute in Warsaw, Poland, served as the PCR positive control. In the amplification procedure, the GeneAmp^®^ PCR System 2700 thermal cycler (Applied Biosystems, Foster City, CA, USA) was used. The following conditions were used: pre-amplification at 94 °C for 3 min, followed by amplification for 30 cycles, each consisting of 94 °C for 30 s, (for annealing temperature see Table 4) for 60 s, 72 °C for 60 s, and a single step of final elongation at 72 °C for 5 min. The obtained amplification products, in the volume of 6 μL, were mixed with Loading Buffer DNA IV (AppliChem, Darmstadt, Germany) and separated in 1.5% agarose gel (Bio-Rad) in 1 × TBE (Bio-Rad), at 9 V/cm for 1.5 h in MINI SUB™ DNA CELL (Bio-Rad) or SUB-CELL^®^ GT (Bio-Rad) along with a 100–3000 bp DNA size marker (Solis BioDyne, Tartu, Estonia). After staining for 30 min in an ethidium bromide solution and subsequent washing for 20 min with deionized water, the gels were visualized in UV light with a Quantity One (Bio-Rad) system, photographed, and stored. The amplified gene identification was done on the basis of fragment size (Table 4). The simultaneous detection of the product with an appropriate size for the strain tested and the PAO1 control strains or ATCC 27853 strain was interpreted as a positive result.

### 4.6. Statistical Methods

Statistical analysis was performed in the StatSoft, Inc.,Cracow, Poland, (2017) STATISTICA 13.1 (data analysis software system, Poland) program using standard chi square test (χ^2^) with α ≤ 0.05 to determine the significance of the differences observed between genes distribution amongst the examined strains, their presence into two distinct examined strains subgroups (MBL-positive and MBL-negative), and with respect to the type of clinical specimen that the strains were isolated from. For the evaluation of gene co-existence and genotype distribution among the examined isolates, the correlation of dependent variables was calculated using the Spearman test (*p* < 0.05).

## 5. Conclusions

A high genetic diversity was noted amongst carbapenem-resistant *P. aeruginosa* strains—most of the strains were found to possess alkaline protease and phospholipase C-coding genes.

The prevalence of virulence genes encoding neuraminidase-1 and elastase B varied with respect to the MBL genes’ presence. It is likely that the decreased virulence of carbapenem-resistant *P. aeruginosa* strains, in some cases, may have resulted from particular gene carriage loss, especially for MBL-producers.

Though carbapenem-resistant *P. aeruginosa* strain virulence is a complex issue, the presence of particular virulence genes, or their genotype composition, might be one of the aspects of their variable infectious and pathogenicity potential, at least in a particular subgroup of the strains.

## Figures and Tables

**Table 1:** The virulence gene incidence and genotype distribution amongst the examined CRPA strains (*n* = 107); (+)—gene presence; (−)—lack of gene; (*)—statistically significant differences (chi^2^ test) observed between *nan1* gene frequency when compared to the rest of the tested gene prevalence amongst the genotypes is indicated (*p* = 0.0134).

Gene/Genotype	A	B	C	D	E	F	G	H	I	*n* = 107	%
*aprA*	+	+	+	+	+	+	+	+	+	107	100.0
*plcN*	+	+	+	+	+	+	+	+	−	106	99.1
*plcH*	+	+	+	+	+	+	+	−	+	106	99.1
*lasB*	+	+	+	−	+	−	−	−	−	98	91.6
*nan2*	+	+	−	+	−	−	+	+	+	94	87.9
nan1 *	−	+	−	−	+	−	+	−	+	40	37.4
*n* = 107	51	36	9	4	2	2	1	1	1		
%	47.7	33.6	8.4	3.7	1.9	1.9	0.9	0.9	0.9		

**Table 2:** The virulence genes distribution amongst the examined strains (*n* = 107) with respect to MBLs carriage—statistically significant differences (chi^2^ test) observed between the tested groups are indicated (* *lasB*—*p* = 0.0038; ** *nan1*—*p* = 0.048).

		Gene					
		*aprA*	*plcN*	*plcH*	lasB *	*nan2*	nan1 **
MBL-positive	*n* = 32	32	32	31	25	27	5
	%	100.0	100.0	96.9	78.1	84.4	15.6
MBL-negative	*n* = 75	75	74	75	73	67	35
	%	100.0	98.7	100.0	97.3	89.3	46.7

**Table 3:** The distribution of the virulence factor genes with respect to specimen type that the strains were isolated from in a number higher than 5.

Clinical Specimen Type	Gene					
	*aprA*	*plcN*	*plcH*	*lasB*	*nan2*	*nan1*
	*n* = 82					
Bronchoalveolar lavage (*n* = 23)	23	23	23	18	18	6
%	100.0	100.0	100.0	78.3	78.3	26.1
Wound swab (*n* = 16)	16	15	15	14	16	6
%	100.0	93.8	93.8	87.5	100.0	37.5
Urine catheterized (*n* = 12)	12	12	12	12	9	2
%	100.0	100.0	100.0	100.0	75.0	16.7
Urine samples (*n* = 12)	12	12	12	12	10	3
%	100.0	100.0	100.0	100.0	83.3	25.0
Blood samples (*n* = 11)	11	11	11	9	11	5
%	100.0	100.0	100.0	81.8	100.0	45.5
Respiratory tract secretion (*n* = 8)	8	8	8	8	7	4
%	100.0	100.0	100.0	100.0	87.5	50.0

**Table 4:** The specification of PCR primers applied in the present study for particular virulence gene detection and amplification product sizes.

Virulence Factor Detected	PCR Primer Name	Primer Sequence 5′→3′	Tm (°C)	Annealing Temperature (°C)	Product Size (bp)
Elastase B	*lasB F*	-GGAATGAACGAAGCGTTCTC-	51.8	50	300
	*lasB R*	-GGTCCAGTAGTAGCGGTTGG-	55.9		
Phospholipase C (H)	*plcH F*	-GAAGCCATGGGCTACTTCAA-	55.1	52	307
	*plcH R*	-AGAGTGACGAGGAGCGGTAG-	58.2		
Phospholipase C (N)	*plcN F*	-GTTATCGCAACCAGCCCTAC-	55.9	53	466
	*plcN R*	-AGGTCGAACACCTGGAACAC-	57.2		
Neuraminidase-1	*nan1 F*	-AGGATGAATACTTATTTTGAT-	42.6	47	1316
	*nan1 R*	-TCACTAAATCCATCTCTGACCCGATA-	56.4		
Neuraminidase-2	*nan2 F*	-ACAACAACGGGGACGGTAT-	51.1	50	1161
	*nan2 R*	-GTTTTGCTGATGCTGGTTCA-	49.7		
Alkaline protease	*aprA F*	-TGTCCAGCAATTCTCTTGC-	48.9	50	1017
	*aprA R*	-CGTTTTCCACGGTGACC-	49.5		

## Data Availability

The data presented in this study are available on request from the corresponding author.
